# Iron-Induced Damage in Cardiomyopathy: Oxidative-Dependent and Independent Mechanisms

**DOI:** 10.1155/2015/230182

**Published:** 2015-03-24

**Authors:** Elena Gammella, Stefania Recalcati, Ilona Rybinska, Paolo Buratti, Gaetano Cairo

**Affiliations:** Department of Biomedical Sciences for Health, University of Milan, Via Mangiagalli 31, 20133 Milan, Italy

## Abstract

The high incidence of cardiomyopathy in patients with hemosiderosis, particularly in transfusional iron overload, strongly indicates that iron accumulation in the heart plays a major role in the process leading to heart failure. In this context, iron-mediated generation of noxious reactive oxygen species is believed to be the most important pathogenetic mechanism determining cardiomyocyte damage, the initiating event of a pathologic progression involving apoptosis, fibrosis, and ultimately cardiac dysfunction. However, recent findings suggest that additional mechanisms involving subcellular organelles and inflammatory mediators are important factors in the development of this disease. Moreover, excess iron can amplify the cardiotoxic effect of other agents or events. Finally, subcellular misdistribution of iron within cardiomyocytes may represent an additional pathway leading to cardiac injury. Recent advances in imaging techniques and chelators development remarkably improved cardiac iron overload detection and treatment, respectively. However, increased understanding of the pathogenic mechanisms of iron overload cardiomyopathy is needed to pave the way for the development of improved therapeutic strategies.

## 1. Introduction

Every cell living under aerobic conditions inevitably and continuously produces reactive oxygen species (ROS), such as anion superoxide (O_2_^•−^) and its dismutation product hydrogen peroxide (H_2_O_2_). Through the iron-catalyzed Fenton reaction, these relatively harmless products are transformed into the highly reactive hydroxyl radical that can attack biological macromolecules and thereby lead to cell and tissue damage if the cellular antioxidant defenses are overwhelmed. At neutral pH most iron precipitates as iron Fe(III) hydroxides; however, in biological environments (e.g., in the cell cytoplasm), Fe(III) can bind low molecular weight compounds like citrate or ATP that are able to keep it soluble but leave one or more iron ligands free to participate in ROS formation [1]. Therefore, this so-called catalytic iron plays an essential role in oxygen radical biochemistry. Interestingly, iron-dependent oxidative injury is not dependent only on the well-known damage that ROS cause to biological macromolecules; a recent study has shown the role of mismetallation, a process in which superoxide and H_2_O_2_ oxidize Fe(II) to Fe(III) and thus displace it from many iron-dependent enzymes that lose their functional capacity [2].

In fact, iron is also essential for a wide range of vital cellular functions such as oxygen transport, energy production, and cell division [1, 3]. For this reason, iron homeostasis is strictly regulated at both the systemic and cellular levels in order to keep iron protein-bound. In the circulation, iron is safely transported by transferrin (Tf), whose binding capacity is normally not fully saturated [4]. Body iron homeostasis is regulated by the interaction of the liver-derived peptide hepcidin and its receptor, the iron exporter ferroportin [3]. Hepcidin binding causes ferroportin internalization and degradation, thereby halting iron efflux. Hepcidin gene expression is controlled by many factors [5], obviously including body iron levels; iron-mediated regulation largely depends on the BMP/SMAD pathway whose activation requires genes like HFE, hemojuvelin, and transferrin receptor 2 (TfR2) that are inactivated in various forms of hereditary hemochromatosis [6].

In the cell, iron regulatory proteins (IRP1 and IRP2) are mRNA-binding proteins that control the synthesis of proteins involved in iron uptake (transferrin receptor, TfR1, and DMT1), storage (ferritin), release (ferroportin), and utilization according to intracellular iron levels. This elegant posttranscriptional mechanism regulates cellular iron balance in order to keep the amount of iron in the redox-active labile iron pool (LIP) low enough to limit toxicity but sufficient for cellular needs [1, 7].

Since no biological mechanisms exist for the excretion of excess iron and hence body iron stores tend to increase with ageing, particularly in females after menopause [8], the maintenance of iron levels within the physiological range could prevent a variety of major diseases and promote longevity [9], as suggested by the increased life span caused by iron restriction in flies and nematodes [10]. Indeed, many of the beneficial effects of polyphenols in these settings may be related to their iron-binding capacity. Interventions aimed at maintaining correct levels of iron (and other micronutrients) in order to achieve healthy ageing and longevity have been recently considered [11].

## 2. Molecular and Cellular Mechanisms Underlying Iron-Dependent Cardiac Damage

### 2.1. Iron and Reactive Oxygen Species Generation in the Heart

All the cell types that constitute the heart may be susceptible to ROS-induced damage, including endothelial cells [12, 13], but in this review we will focus on cardiomyocytes that are particularly susceptible to ROS-mediated damage. In fact, on the one hand, given the heart's high need of energy, cardiomyocytes are rich in mitochondria and consume large amounts of oxygen; on the other hand, cardiomyocytes have low levels of antioxidant enzymes [14]. However, direct reaction between oxygen and biomolecules is slow in the absence of a catalyst, and iron is the more potent catalyst of oxidative stress. Therefore, when LIP expansion occurs, oxidative stress can affect cardiac functions. Increasing the synthesis of ferritin, the cytosolic protein that safely stores iron, represents a response to iron-mediated oxidative stress [15]. In the heart, synthesis of ferritin, which is enriched in H subunit [16] and sequestrates redox-active iron, is at the basis of an iron-based mechanism of ischemic preconditioning that protects cardiac cells from iron-mediated oxidative damage associated with ischemia-reperfusion injury [17]. However, almost 20 years ago it has been shown that liver ferritin can act both as an antioxidant and a prooxidant in a time-dependent manner. Oxidative stress caused by glutathione depletion [18] or postischemic reperfusion [19] first induced ferritin degradation and consequent LIP expansion that subsequently activated ferritin expression, thus limiting the prooxidant challenge of iron. A similar mechanism has been recently described in the context of myocardial protection by ischemic preconditioning; proteasomal ferritin degradation leads to cytosolic iron release, thus triggering de novo apoferritin synthesis and sequestration of reactive iron [20].

### 2.2. Altered Cardiac Iron Balance

Since under normal conditions Tf-bound iron is not limiting, the uptake of iron into the cells is controlled by the regulated expression of TfR1 [21]. Therefore, iron overload is observed only when the binding capacity of Tf is saturated and non-transferrin-bound iron (NTBI) or labile plasma iron (LPI), the fraction of NTBI that is able to penetrate into the cells and is chelatable [22], is formed. Although NTBI is clearly implicated in tissue siderosis, how it enters the cell remains undefined, largely because of the incomplete characterization of the iron species involved, with variable composition under different pathological conditions and changeable association with macromolecules. NTBI has been claimed to be associated with various plasma components, such as citrate, phosphates, and proteins [23]. After extracellular reduction to ferrous iron by a cell-surface reductase like Dcytb, NTBI enters into cells mainly through divalent cation transporters such as DMT1 and ZIP14 [24, 25]. However, the pathway of NTBI uptake by cardiomyocytes appears to be mainly mediated by Ca channels, either L-type or T-type [26, 27].

Iron's toxicity within cells arises from its capacity to catalyse the production of ROS that cause lipid peroxidation and organelle damage [28], which leads to cell death and fibrosis and ultimately impaired systolic and diastolic function. A recent study in a mouse cardiomyocyte cell line showed that L-type channels are the more important mediators of iron uptake [29]. The import of ferrous iron by L-type channels, with consequent higher ROS production, may inhibit calcium influx, thereby affecting cardiac excitation-contraction coupling that is highly sensitive to changes in cellular redox state (reviewed by [30]). This in turn will lead to the impaired systolic and diastolic function that is a characteristic of iron-overload cardiomyopathy. Interestingly, a recent paper showed that iron accumulation mediated by the iron-binding protein Lipocalin-2 leads to cardiomyocyte apoptosis and heart remodeling [31].

Exporting excess iron may represent a way to counterbalance NTBI influx, and indeed it has been shown that heart overload induces ferroportin mRNA levels in hemojuvelin-deficient mice [32]. Similarly, marked upregulation of cardiomyocyte ferroportin in mice lacking hepcidin was protective against systemic iron overload [13]. In many conditions leading to iron-mediated cardiomyopathy, such as *β* thalassemia, hepcidin levels are paradoxically low despite the induction associated with body iron overload because erythropoiesis-dependent downregulation prevails [33]. Therefore, under these settings, low levels of circulating hepcidin may concur with the transcriptional upregulation [32] to enhance ferroportin expression and export activity, thereby helping the heart avoid iron accumulation, at least until a certain threshold is reached [34].

### 2.3. Iron- and Anthracycline-Induced Cardiotoxicity

Iron can also play a relevant role in cardiotoxicity induced by other agents, as in the case of anthracycline-dependent cardiotoxicity. Doxorubicin (DOX) belongs to the family of antitumor anthracyclines that are among the most effective agents in a number of neoplastic diseases. Unfortunately, the clinical use of DOX is limited by the possible development of cardiomyopathy and congestive heart failure. Given the tight relationship between the amount of DOX accumulated in the heart and the incidence of cardiac events, the cumulative dose of 500 mg/m^2^ DOX should not be exceeded; respecting this threshold reduces considerably the incidence of cardiac events but can preclude successful completion of chemotherapy [35].

Many observations indicate that iron plays a role in DOX cardiotoxicity, such as the higher susceptibility to DOX-dependent cardiac damage found in mice lacking HFE, a model that mimics the iron overload found in human hereditary hemochromatosis [36], and the association of myocardial injury with the frequency of HFE gene mutations in survivors of childhood acute lymphoblastic leukemia who were exposed to anthracyclines [37]. Moreover, convincing evidence showed that iron chelation effectively protects against cardiotoxicity in patients and animal models [35]. While several studies demonstrated that iron overload exacerbates the cardiotoxic effects of the drug, the underlying molecular mechanisms remain to be fully understood. Since anthracyclines degradation, by virtue of reductive activation of the quinone moiety of DOX by a number of reductases, eventually results in the formation of O_2_^•−^ and H_2_O_2_, amplification of iron-catalyzed formation of free radicals may represent an obvious mechanism at the basis of the role of iron in DOX cardiotoxicity. However, while anthracycline cardiotoxicity is relieved by iron chelators like dexrazoxane, none of several antioxidants offered protection against chronic cardiotoxicity in clinical settings [35, 38, 39]. How the interaction between anthracyclines and iron damages heart cells is still unclear but may be mediated not by DOX-iron interactions but by profound interference with proteins of iron metabolism, thereby ultimately leading to dysregulation of fundamental cellular events. The major processes linking iron and anthracycline metabolism involve ferritin and IRPs. We showed that DOX can inactivate both IRPs, an event that disrupts cellular iron homeostasis, thereby triggering serious pathologic consequences (reviewed in [35]). Regarding ferritin, while initial in vitro experiments suggested that ferritin may represent a source of catalytically active iron released upon O_2_^−^ formation [40], recent data provided by in vivo studies indicate that ferritin synthesis may rather be a defensive mechanism to limit the amount of iron available for ROS production in the heart (reviewed in [41]). Since it is well known that cardiac mitochondria are preferential targets of anthracyclines, attention has been recently focused on the role of mitochondrial ferritin (FtMt), a recently identified ferritin type that accumulates specifically in the mitochondria which, thanks to its ferroxidase activity, actively sequesters iron and reduces iron-mediated oxidative damage. Two recent papers highlighted the role of FtMt in these settings: FtMt is induced in the heart of DOX-treated mice [42] and higher susceptibility to DOX-dependent oxidative cardiac damage was found in FtMt-deficient mice [43]. These data, together with the recent demonstration that the expression of ABCB8, which is involved in iron export out of the mitochondria, is key to modulate heart sensitivity to DOX [42], underline the importance of mitochondrial iron availability for anthracycline cardiotoxicity.

The iron chelator dexrazoxane is the only agent protecting against DOX cardiotoxicity; however, the mechanisms underlying its protective role, which may include activation of hypoxia inducible factor [44], remain to be fully established [41]. A recent study showed beneficial effects of lovastatin against DOX-induced delayed cardiotoxicity in mice [45]. Statin drugs have pleiotropic effects independent of their capacity to lower cholesterol. In particular, it has been shown that they influence the expression of proteins of iron metabolism like heme oxygenase and hepcidin (discussed by [46]). Further studies are needed to assess the usefulness of statins for the prevention of iron-mediated cardiotoxicity.

### 2.4. Iron Maldistribution

Iron-mediated cell damage does not occur only under conditions of systemic iron overload. Indeed, recent evidence showed that iron maldistribution among organs or tissues and also among cellular compartments can affect cell integrity and life [47]. An example of the first type of incorrect iron distribution is the retention of iron in the reticuloendothelial system that is typically observed under inflammatory conditions and contributes to the development of anemia of chronic disease. On the other hand, the heart is particularly affected by maldistribution within cardiac cells. Mitochondria appear to be the organelles primarily affected by subcellular regional iron accumulation, and this fact, along with their high production of ROS, lays the foundation for oxidative damage. Moreover, disruption of iron homeostasis in mitochondria may play a role in the ROS-mediated mitochondrial decay that accompanies aging [10]. Mitochondrial iron levels are strictly controlled, as the dual role of iron (necessary but dangerous) is particularly evident in this organelle that is involved in heme synthesis and iron-sulfur cluster assembly. This is in line with recent evidence showing that iron is a pivotal regulator of mitochondrial biogenesis [48].

Under iron overload conditions, iron accumulation in mitochondria may be the result of very efficient and fast iron transfer to these organelles. In fact, it has been demonstrated that cardiac mitochondria rapidly acquire NTBI, commonly present in the plasma of iron-loaded patients [49]. In mice injected with iron dextran, iron accumulation in the heart was accompanied by damage to mitochondrial DNA and impaired synthesis of mitochondrial respiratory chain subunits, eventually leading to respiratory dysfunction and cardiomyopathy [50]. Moreover, mitochondria-specific iron overload can result from impaired utilization of iron for the synthesis of heme, such as in sideroblastic anemia, or Fe-S clusters, such as in Friedriech ataxia (FRDA) [51]. Defective iron sulfur cluster biogenesis due to frataxin deficiency in FRDA leads to mitochondrial iron accumulation by means of still not fully understood mechanisms and is accompanied by progressive cardiomyopathy [52]. Other conditions characterized by alterations in different genes involved in Fe-S cluster biogenesis are accompanied by mitochondrial iron accumulation. Examples are the mutations in the scaffold protein ISCU in Swedish myopathy or in the ABCB7 gene in X-linked sideroblastic anemia with ataxia and the mutation in glutaredoxin GLRX5 identified in a patient presenting sideroblastic anemia (reviewed in [53]). Interestingly, recent data (discussed in detail in [52]) seem to suggest that, despite mitochondrial iron excess, the cell and the organelles may face iron deprivation, as the accumulated iron is not biologically available.

Other pathogenetic mechanisms leading to mitochondrial iron accumulation may be related to defective export or unregulated import. The ATP-binding cassette transporter ABCB8 exports iron out of mitochondria and its absence leads to mitochondrial iron accumulation, oxidative stress, cardiomyopathy, and higher susceptibility to DOX-induced cardiotoxicity [54]. In this context, recent findings indicate a role for proteins belonging to the NEET family that are involved in the transfer of cluster/iron to mitochondria. NEET proteins influence mitochondrial iron levels and hence may play an important role in the control of cellular iron metabolism and ROS homeostasis [55].

The role of correct mitochondrial iron management is highlighted by the existence of MtFt that readily sequesters iron in these organelles (see above). By so doing, FtMt protects mitochondria against iron-dependent oxidative damage and also modifies cellular iron distribution by attracting iron from the cytosol to mitochondria [15]. FtMt is highly expressed in the heart and protects cardiac mitochondria against DOX-induced oxidative damage [43].

Reducing mitochondrial iron may protect the heart through inhibition of oxidative stress. However, mitochondria-targeted iron chelators should be used in order to avoid (or not increase) systemic iron deficiency. This approach has been already pursued, as mobilization and redistribution of mitochondrial iron was reported with the use of deferiprone [56] (see below).

### 2.5. Progression from Cardiomyocyte Injury to Heart Disease

Although production of hydroxyl radical and lipid peroxidation are important in the initiation of iron-overload cardiomyopathy, additional mechanisms involving apoptosis and fibrosis can account for its complex pathophysiology that leads to heart failure [27, 57, 58].

For example, it has been shown that thromboxane A2 mediates iron-overload cardiomyopathy through the TNF-*α*-associated calcineurin-NFAT signaling pathway [59]. Other studies have identified the importance of proinflammatory mediators, including TNF-*α*, MCP-1, and IL-6, expressed within the myocardium in response to high blood pressure, oxidative stress, and tissue injury, resulting in cardiac remodeling that includes cardiomyocyte apoptosis and cardiac fibrosis (reviewed in [30]). These findings indicate that macrophage-cardiomyocyte interactions play a key role in cardiac inflammation and subsequent fibrosis found in iron overload cardiomyopathy. Indeed, prolonged increase in myocardial fibrotic activity results in stiffening of the heart and is an indicator of adverse outcomes related to systolic and diastolic dysfunction, as well as arrhythmogenesis. Regarding fibrosis, matrix metalloproteinases (MMPs), that are important for pathophysiological tissue repair, appear to be involved in cardiac remodeling. The finding that an iron-binding protein like Lipocalin-2 is induced in a porcine model of heart failure and associates with MMP-9 [60] suggests that iron may also directly influence heart fibrotic response.

## 3. Preclinical Animal Models of Cardiac Iron Overload

Recent advances in understanding the pathophysiology of iron-dependent cardiomyopathy have been made possible also by the availability of animal models. Several studies used parenteral iron administration in mice (see e.g., [61]), but also in gerbils [62], to investigate cardiac iron overload and damage. However, in this context the cellular distribution of excess iron, which preferentially targets macrophages rather than cardiomyocytes, does not closely reproduce human heart pathology.

In addition to mouse models of *β* thalassemia, which is the secondary iron overload condition more closely linked to cardiac iron overload [63], animal models involving ablation of genes that are inactivated in hereditary hemochromatosis also represent useful and validated models of body (and heart) iron overload. Mouse models involving HFE [64], hemojuvelin [32, 65], hepcidin [66], and transferrin receptor 2 (TfR2) [64] inactivation lead to iron loading phenotypes ranging from mild to severe. Cardiac iron accumulation is slower than hepatic iron loading, but, despite the more gradual accumulation, heart iron levels and distribution in mice lacking hemojuvelin are similar to humans. However, while in human hemochromatosis long-term deposition of iron in the heart can lead to cardiomyopathy, such pathological consequence of iron overload is more difficult to obtain in corresponding mouse models. Recently, it has been shown that cardiomyocyte-targeted deletion of ferroportin leads to increased cardiomyocyte iron deposition and severely impaired cardiac function despite a moderate degree of iron loading [13]. This study elegantly showed that the effects of iron depend on the site of accumulation.

Despite the divergence in cardiac phenotypes induced by iron overload in humans and mice, these models also proved to be very useful to test novel therapeutic approaches to treat cardiac iron overload (see below).

## 4. Clinical Conditions Involving Cardiac Iron Overload

It is well recognized that systemic iron overload conditions, in which the iron chelating capacity of our blood (i.e., Tf) is exceeded, are associated with cardiomyopathy [67]. However, it should be remarked that, in line with the idea that the organism requires a balanced amount of iron, also iron deficiency is frequently found in patients with heart failure and represents a serious comorbidity associated with poor clinical outcomes [68]. Cardiac iron overload is commonly associated with hemochromatosis and disorders requiring repeated blood transfusion (see below); however, the adverse effects of iron overload are also found in patients undergoing hematopoietic stem cell transplantation. In these settings, NTBI formation occurs upon initiation of the conditioning regimen until engraftment, due to the inhibition of iron utilization by the erythroid compartment [69]. The clinical dysfunctions of iron overload cardiomyopathy have been excellently covered elsewhere [27] and will not be treated in detail here.

### 4.1. Primary Iron Overload

In HFE-linked hereditary hemochromatosis patients, iron overload first affects the liver, but cardiac problems (arrhythmias and heart failure) are also found, although in a low percentage of cases [70]. In addition, ventricular systolic function may be normal at rest but compromised under exercise [71]. Moreover, in juvenile hereditary hemochromatosis, which shows a more severe phenotype, death from intractable heart failure is common [72]. Diastolic dysfunction and arrhythmias that are present initially can progress to dilated cardiomyopathy. Early treatment (usually by phlebotomy) is effective but the average survival is less than a year in untreated patients with severe cardiac impairment [73]. Remarkably, the iron concentration in the heart shows heterogeneous distribution and is significantly lower than in the liver, thus confirming the high susceptibility of cardiac cells to iron-mediated oxidative injury.

### 4.2. Secondary Iron Overload

Secondary iron overload is mainly observed in association with transfusion-dependent diseases [67, 74]. Because humans lack any effective means to excrete excess iron, long-term transfusion alone inexorably produces the clinical problem of iron overload in patients needing repeated transfusion (e.g., thalassemia, Blackfan-Diamond anemia, aplastic anemia, sideroblastic anemia, and myelodysplasia). With continued transfusion, macrophages and hepatocytes can no longer retain all the surplus iron that then enters plasma in amounts that exceed the transport capacity of circulating Tf, and NTBI develops. Transfusional iron overload affects particularly patients with inherited hemoglobinopathies, which are the most common single-gene disorders in humans, as all thalassemia major patients and 20% of those with sickle cell disease are transfusion-dependent [75].

While the necessity of transfusion for severe anemia is undisputed, it is clear that the risk of transfusion-dependent iron overload should be carefully considered. Patients with low-grade myelodysplasia who have sufficient longevity may experience the deleterious effects of cardiac iron overload that adversely impact survival in these patients. Treatment with iron chelators (see below) avoids cardiac dysfunction in these settings, although the beneficial effect of chelation in preventing cardiac dysfunction may not be linked only to the removal of excess tissue iron [76].

## 5. Detection of Cardiac Iron Overload

Several diagnostic methods have been developed in order to detect myocardial iron overload as early as possible, thereby averting a process that can lead to cardiomyopathy, heart failure, and ultimately death. These include evaluation of serum ferritin levels, electrocardiography (ECG) echocardiography, and cardiac magnetic resonance imaging T2^*^ (cMRI-T2^*^). While ECG, echocardiography, and serum ferritin levels are very easy and cheap methods, they are either not specific for cardiac overload or not enough sensitive for the detection of iron in the heart. Since iron is able to reduce the magnetic resonance signal, the parameter called T2^*^ measures the amount of iron in heart as the loss of signal in iron-loaded tissue. cMRI-T2^*^, which is based on the evaluation of T2^*^, is the most efficient diagnostic technique for the evaluation of cardiac iron overload [77, 78]. T2^*^ values lower than 20 ms (physiological value) indicate presence of iron overload and, as T2^*^ changes according to the concentration of iron, values lower than 10 ms indicate the presence of severe iron overload with high risk of developing cardiac dysfunction within 1 year [77, 79]. It should be pointed out that cMRI is an efficient method also to evaluate the efficacy of iron chelator therapies. In addition, the early detection of cardiac iron overload and associated cardiac dysfunction by means of cMRI-T2^*^ allows more time for reversal through iron chelation therapy.

## 6. Treatment of Cardiac Iron Overload

The treatment of cardiac iron overload is mainly based on the use of iron chelators. The majority of data regarding the treatment of transfusional iron overload are derived from the thalassemia population [80]. However, the use of iron chelators resulted to be efficient in reducing iron overload and improving survival also in transfusion-dependent patients with myelodysplastic syndrome [76].

The chelators presently approved by EU and FDA for clinical use include deferoxamine, deferiprone, and deferasirox [81]. Introduction of deferoxamine 40 years ago dramatically changed the cure and life expectancy of patients with cardiac iron overload, in particular thalassemic patients. Long-term therapy with deferoxamine is associated with a reduction in cardiac complications and improved survival. However, deferoxamine is a lipophobic molecule with limited capacity to chelate intracellular iron and has several side effects. Moreover, adherence to deferoxamine therapy is a challenge because it has a short half-life and hence has to be usually administered by prolonged subcutaneous infusion [82]. Despite recent findings showing that new molecules may be more effective for removing heart iron [83], deferoxamine is still one of the most widely used iron chelators and is the recommended first-line therapy for iron overload in people with thalassaemia major [84].

The limits of deferoxamine prompted the search for alternative drugs and two oral iron chelators, deferiprone and deferasirox, are now commonly used.

Deferiprone is a lipophilic molecule with high chelating capacity for intracellular iron that can be given orally and it is quickly absorbed. These properties enable its prompt entry into cells, including those of cardiac tissue [85]. Studies have demonstrated reduced cardiac morbidity and mortality and lower myocardial iron deposition among patients treated with deferiprone. Side effects include reduced number of neutrophils [86].

Also deferasirox is orally administered once a day, due to its long half-life. It is a lipophilic, low molecular weight molecule that is able to chelate intracellular iron as well as iron in the blood, thereby preventing cardiac iron accumulation. This effect was recently confirmed in the prospective, multicentre study of deferasirox in transfusion-dependent thalassemic patients with evidence of myocardial iron overload without cardiac dysfunction [87].

The choice of the best therapy depends on the severity of cardiac siderosis, the estimated total body iron burden, the presence of organ dysfunction, and the rate of transfusion therapy. Iron is accumulated in (and removed from) different organs at different rates, and hepatic iron stores usually change more rapidly than cardiac iron levels, so both hepatic and cardiac iron must be measured to optimize chelation. In addition, a recent study showed that deferiprone has greater efficacy in removing myocardial iron burden, whereas deferoxamine was particularly effective against liver iron deposition [83].

We will not address here the extensive literature regarding the clinical efficacy of the different chelating therapies using the various chelators, either alone or in combination (see [86] for a review). However, we will briefly examine the chelating properties of the various molecules in relation to their susceptibility towards redox cycling.

To maximize the thermodynamic stability of the iron(III) ligand complex, all the six iron atoms should be bound to donor atoms of the chelator to form a stable inert complex. Ligands can be structurally classified according to the number of donor atoms that each molecule possesses, that is, bidentate (e.g., deferiprone, least stable), tridentate (e.g., deferasirox), and hexadentate (e.g., deferoxamine, most stable). Low stability increases the risk of the participation of incomplete iron-chelate complexes in the generation of harmful free radicals. As a consequence, only with dosages high enough (>10 *μ*M) to reach a 3 : 1 molar ratio of bidentate chelator to iron, stable iron-coordinate complexes and efficient and safe scavenging of excess iron will be achieved [88, 89]. Interestingly, this capacity of the iron-deferiprone complex to donate iron to competing ligands at relatively low concentrations has been exploited to develop combination therapies in which deferiprone is used together with a high affinity hexadentate chelator such as deferoxamine. In this context, the deferiprone-iron complex will readily donate iron to the kinetically more stable deferoxamine (shuttle effect) [90]. Clinical studies indicated that such approach minimized side effects (by virtue of lower doses) and was effective at increasing iron removal and survival. An excellent overview of combination iron chelation therapy has been presented [91].

Given the role of L-type and T-type Ca channels in iron import into cardiomyocytes (see above), calcium channel blockers were considered for cardiac iron overload treatment. The L-type calcium channel blockers amlodipine and verapamil proved to be effective in treating heart iron overload in a validated mouse model [92, 93] and the T-type calcium blocker efonidipine improved cardiac function in a mouse model of thalassemia [94]. Remarkably, amlodipine was successfully used in patients [95, 96].

In view of the role of oxidative damage in cardiac iron overload, another potential therapy is represented by antioxidants. Indeed, it has been demonstrated that taurine supplementation reduced oxidative stress in the heart of iron overloaded mice, thereby preserving cardiovascular function and improving survival [57]. In line with the tight relationship between iron and oxidative stress, iron chelators and prochelators are presently considered for their capacity to protect cardiac cells against injury induced by ROS [97].

## 7. Conclusions

**Figure 1:**
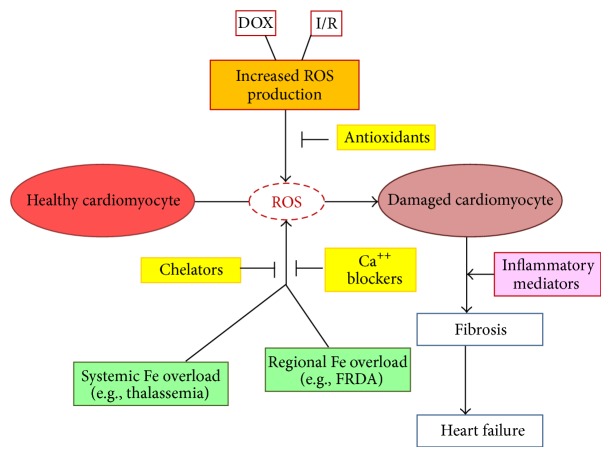
Roles of iron in the pathologic progression leading to cardiac dysfunction. Excess iron, derived from either systemic overload (e.g., in thalassemia) or mislocalization (e.g., in FRDA), can directly catalyze ROS formation. Alternatively, iron may function as cofactor of other damaging agents, such as the cardiotoxic drug doxorubicin, or events, like postischemic reperfusion, and amplify their oxidative-dependent cardiotoxic effect. As described in the text, iron may also influence inflammatory events and repair processes, thereby fueling the progression of cardiomyopathy. The possible therapeutic interventions are also highlighted.

The role of iron in the pathogenesis of cardiac damage is undisputed, but the underlying molecular mechanisms remain incompletely understood. However, it is possible to anticipate that our increased understanding of the regulatory network that controls iron homeostasis may lead to a better management of cardiac iron overload. The studies highlighted in this review have shown that the role of iron can be manifold. While early studies pointed to a role as amplifier of the free radical generation initiated by redox cycling, more recent evidence suggests that the cardiotoxic role of iron should not be restricted to the oxidative stress scenario. Iron can act in concert with other damaging agents or events and amplify their cardiotoxic effect (Figure 1). Another important aspect is the site of iron accumulation, both among different heart cells and within the cardiomyocyte, as not only iron overload but also mislocalization can lead to cardiomyopathy. Defects in iron handling (particularly in the mitochondria) may contribute to heart damage through ROS-mediated mechanisms but also through disruption of Fe/S cluster and/or heme synthesis. Moreover, iron not only can play a role in cardiomyocyte damage but also influence the mechanisms leading to the progression of cardiac dysfunction, as iron-dependent expression of macrophage-derived inflammatory mediators may influence fibrosis and the progression of cardiomyopathy. Exploring more deeply the pathogenic mechanisms of iron overload cardiomyopathy will reveal useful insights for the development of novel therapeutic strategies.

## References

[1] Pantopoulos K., Porwal S. K., Tartakoff A., Devireddy L. (2012). Mechanisms of mammalian iron homeostasis. *Biochemistry*.

[2] Imlay J. A. (2014). The mismetallation of enzymes during oxidative stress. *The Journal of Biological Chemistry*.

[3] Hentze M. W., Muckenthaler M. U., Galy B., Camaschella C. (2010). Two to tango: regulation of mammalian iron metabolism. *Cell*.

[4] Garrick M. D. (2011). Human iron transporters. *Genes & Nutrition*.

[5] Meynard D., Babitt J. L., Lin H. Y. (2014). The liver: conductor of systemic iron balance. *Blood*.

[6] Ganz T., Nemeth E. (2012). Hepcidin and iron homeostasis. *Biochimica et Biophysica Acta—Molecular Cell Research*.

[7] Recalcati S., Minotti G., Cairo G. (2010). Iron regulatory proteins: from molecular mechanisms to drug development. *Antioxidants and Redox Signaling*.

[8] Zacharski L. R., Ornstein D. L., Woloshin S., Schwartz L. M. (2000). Association of age, sex, and race with body iron stores in adults: analysis of NHANES III data. *American Heart Journal*.

[9] Toyokuni S. (2011). Iron as a target of chemoprevention for longevity in humans. *Free Radical Research*.

[10] Xu J., Marzetti E., Seo A. Y., Kim J.-S., Prolla T. A., Leeuwenburgh C. (2010). The emerging role of iron dyshomeostasis in the mitochondrial decay of aging. *Mechanisms of Ageing and Development*.

[11] Mocchegiani E., Costarelli L., Giacconi R., Piacenza F., Basso A., Malavolta M. (2012). Micronutrient (Zn, Cu, Fe)-gene interactions in ageing and inflammatory age-related diseases: implications for treatments. *Ageing Research Reviews*.

[12] Chen Y.-R., Zweier J. L. (2014). Cardiac mitochondria and reactive oxygen species generation. *Circulation Research*.

[13] Lakhal-Littleton S., Wolna M., Carr C. A. (2015). Cardiac ferroportin regulates cellular iron homeostasis and is important for cardiac function. *Proceedings of the National Academy of Sciences of the United States of America*.

[14] Doroshow J. H., Locker G. Y., Myers C. E. (1980). Enzymatic defenses of the mouse heart against reactive oxygen metabolites: alterations produced by doxorubicin. *Journal of Clinical Investigation*.

[15] Arosio P., Levi S. (2010). Cytosolic and mitochondrial ferritins in the regulation of cellular iron homeostasis and oxidative damage. *Biochimica et Biophysica Acta—General Subjects*.

[16] Cairo G., Rappocciolo E., Tacchini L., Schiaffonati L. (1991). Expression of the genes for the ferritin H and L subunits in rat liver and heart. Evidence for tissue-specific regulations at pre- and post-translational levels. *Biochemical Journal*.

[17] Chevion M., Leibowitz S., Aye N. N. (2008). Heart protection by ischemic preconditioning: a novel pathway initiated by iron and mediated by ferritin. *Journal of Molecular and Cellular Cardiology*.

[18] Cairo G., Tacchini L., Pogliaghi G., Anzon E., Tomasi A., Bernelli-Zazzera A. (1995). Induction of ferritin synthesis by oxidative stress: transcriptional and post-transcriptional regulation by expansion of the ‘free’ iron pool. *The Journal of Biological Chemistry*.

[19] Tacchini L., Recalcati S., Bernelli-Zazzera A., Cairo G. (1997). Induction of ferritin synthesis in ischemic-reperfused rat liver: analysis of the molecular mechanisms. *Gastroenterology*.

[20] Bulvik B. E., Berenshtein E., Meyron-Holtz E. G., Konijn A. M., Chevion M. (2012). Cardiac protection by preconditioning is generated via an iron-signal created by proteasomal degradation of iron proteins. *PLoS ONE*.

[21] Frazer D. M., Anderson G. J. (2014). The regulation of iron transport. *BioFactors*.

[22] Cabantchik Z. I. (2014). Labile iron in cells and body fluids: physiology, pathology, and pharmacology. *Frontiers in Pharmacology*.

[23] Hider R. C. (2002). Nature of nontransferrin-bound iron. *European Journal of Clinical Investigation*.

[24] Liuzzi J. P., Aydemir F., Nam H., Knutson M. D., Cousins R. J. (2006). Zip14 (Slc39a14) mediates non-transferrin-bound iron uptake into cells. *Proceedings of the National Academy of Sciences of the United States of America*.

[25] Ludwiczek S., Theurl I., Muckenthaler M. U. (2007). Ca^2+^ channel blockers reverse iron overload by a new mechanism via divalent metal transporter-1. *Nature Medicine*.

[26] Oudit G. Y., Sun H., Trivieri M. G. (2003). L-type Ca^2+^ channels provide a major pathway for iron entry into cardiomyocytes in iron-overload cardiomyopathy. *Nature Medicine*.

[27] Murphy C. J., Oudit G. Y. (2010). Iron-overload cardiomyopathy: pathophysiology, diagnosis, and treatment. *Journal of Cardiac Failure*.

[28] Brissot P., Ropert M., Le Lan C., Loréal O. (2012). Non-transferrin bound iron: a key role in iron overload and iron toxicity. *Biochimica et Biophysica Acta—General Subjects*.

[29] Rose R. A., Sellan M., Simpson J. A. (2011). Iron overload decreases Ca_V_1.3-dependent L-type Ca^2+^ currents leading to bradycardia, altered electrical conduction, and atrial fibrillation. *Circulation: Arrhythmia and Electrophysiology*.

[30] Cheng C.-F., Lian W.-S. (2013). Prooxidant mechanisms in iron overload cardiomyopathy. *BioMed Research International*.

[31] Xu G., Ahn J., Chang S. (2012). Lipocalin-2 induces cardiomyocyte apoptosis by increasing intracellular iron accumulation. *The Journal of Biological Chemistry*.

[32] Brewer C. J., Wood R. I., Wood J. C. (2014). mRNA regulation of cardiac iron transporters and ferritin subunits in a mouse model of iron overload. *Experimental Hematology*.

[33] Kautz L., Nemeth E. (2014). Molecular liaisons between erythropoiesis and iron metabolism. *Blood*.

[34] Noetzli L. J., Carson S. M., Nord A. S., Coates T. D., Wood J. C. (2008). Longitudinal analysis of heart and liver iron in thalassemia major. *Blood*.

[35] Minotti G., Menna P., Salvatorelli E., Cairo G., Gianni L. (2004). Anthracyclines: molecular advances and pharmacologic developments in antitumor activity and cardiotoxicity. *Pharmacological Reviews*.

[36] Miranda C. J., Makui H., Soares R. J. (2003). Hfe deficiency increases susceptibility to cardiotoxicity and exacerbates changes in iron metabolism induced by doxorubicin. *Blood*.

[37] Lipshultz S. E., Lipsitz S. R., Kutok J. L. (2013). Impact of hemochromatosis gene mutations on cardiac status in doxorubicin-treated survivors of childhood high-risk leukemia. *Cancer*.

[38] Octavia Y., Tocchetti C. G., Gabrielson K. L., Janssens S., Crijns H. J., Moens A. L. (2012). Doxorubicin-induced cardiomyopathy: from molecular mechanisms to therapeutic strategies. *Journal of Molecular and Cellular Cardiology*.

[39] Stěrba M., Popelová O., Vávrová A. (2013). Oxidative stress, redox signaling, and metal chelation in anthracycline cardiotoxicity and pharmacological cardioprotection. *Antioxidants and Redox Signaling*.

[40] Thomas C. E., Aust S. D. (1986). Reductive release of iron from ferritin by cation free radicals of paraquat and other bipyridyls. *The Journal of Biological Chemistry*.

[41] Gammella E., Maccarinelli F., Buratti P., Recalcati S., Cairo G. (2014). The role of iron in anthracycline cardiotoxicity. *Frontiers in Pharmacology*.

[42] Ichikawa Y., Ghanefar M., Bayeva M. (2014). Cardiotoxicity of doxorubicin is mediated through mitochondrial iron accumulation. *Journal of Clinical Investigation*.

[43] Maccarinelli F., Gammella E., Asperti M. (2014). Mice lacking mitochondrial ferritin are more sensitive to doxorubicin-mediated cardiotoxicity. *Journal of Molecular Medicine*.

[44] Spagnuolo R. D., Recalcati S., Tacchini L., Cairo G. (2011). Role of hypoxia-inducible factors in the dexrazoxane-mediated protection of cardiomyocytes from doxorubicin-induced toxicity. *British Journal of Pharmacology*.

[45] Henninger C., Huelsenbeck S., Wenzel P. (2015). Chronic heart damage following doxorubicin treatment is alleviated by lovastatin. *Pharmacological Research*.

[46] Mascitelli L., Goldstein M. R. (2012). Might the beneficial effects of statin drugs be related to their action on iron metabolism?. *Quarterly Journal of Medicine*.

[47] Cabantchik Z. I., Munnich A., Youdim M. B., Devos D. (2013). Regional siderosis: a new challenge for iron chelation therapy. *Frontiers in Pharmacology*.

[48] Rensvold J. W., Ong S.-E., Jeevananthan A., Carr S. A., Mootha V. K., Pagliarini D. J. (2013). Complementary RNA and protein profiling identifies iron as a key regulator of mitochondrial biogenesis. *Cell Reports*.

[49] Shvartsman M., Kikkeri R., Shanzer A., Cabantchik Z. I. (2007). Non-transferrin-bound iron reaches mitochondria by a chelator-inaccessible mechanism: biological and clinical implications. *American Journal of Physiology—Cell Physiology*.

[50] Gao X., Qian M., Campian J. L. (2010). Mitochondrial dysfunction may explain the cardiomyopathy of chronic iron overload. *Free Radical Biology and Medicine*.

[51] Payne R. M. (2011). The heart in friedreich's ataxia: basic findings and clinical implications. *Progress in Pediatric Cardiology*.

[52] Martelli A., Puccio H. (2014). Dysregulation of cellular iron metabolism in Friedreich ataxia: from primary iron-sulfur cluster deficit to mitochondrial iron accumulation. *Frontiers in Pharmacology*.

[53] Beilschmidt L. K., Puccio H. M. (2014). Mammalian Fe-S cluster biogenesis and its implication in disease. *Biochimie*.

[54] Ichikawa Y., Ghanefar M., Bayeva M. (2014). Cardiotoxicity of doxorubicin is mediated through mitochondrial iron accumulation. *The Journal of Clinical Investigation*.

[55] Tamir S., Paddock M. L., Darash-Yahana-Baram M. (2014). Structure-function analysis of NEET proteins uncovers their role as key regulators of iron and ROS homeostasis in health and disease. *Biochimica et Biophysica Acta—Molecular Cell Research*.

[56] Sohn Y.-S., Breuer W., Munnich A., Cabantchik Z. I. (2008). Redistribution of accumulated cell iron: a modality of chelation with therapeutic implications. *Blood*.

[57] Oudit G. Y., Trivieri M. G., Khaper N. (2004). Taurine supplementation reduces oxidative stress and improves cardiovascular function in an iron-overload murine model. *Circulation*.

[58] Wang Y., Wu M., Al-Rousan R. (2011). Iron-induced cardiac damage: role of apoptosis and deferasirox intervention. *Journal of Pharmacology and Experimental Therapeutics*.

[59] Lin H., Li H.-F., Lian W.-S. (2013). Thromboxane A2 mediates iron-overload cardiomyopathy in mice through calcineurin-nuclear factor of activated t cells signaling pathway. *Circulation Journal*.

[60] Kiczak L., Tomaszek A., Bania J. (2014). Matrix metalloproteinase 9/neutrophil gelatinase associated lipocalin/tissue inhibitor of metalloproteinasess type 1 complexes are localized within cardiomyocytes and serve as a reservoir of active metalloproteinase in porcine female myocardium. *Journal of Physiology and Pharmacology*.

[61] Moon S. N., Han J. W., Hwang H. S. (2011). Establishment of secondary iron overloaded mouse model: evaluation of cardiac function and analysis according to iron concentration. *Pediatric Cardiology*.

[62] Otto-Duessel M., Brewer C., Gonzalez I., Nick H., Wood J. C. (2008). Safety and efficacy of combined chelation therapy with deferasirox and deferoxamine in a gerbil model of iron overload. *Acta Haematologica*.

[63] Sanyear C., Butthep P., Nithipongvanich R. (2013). Cardiomyocyte ultrastructural damage in *β*-thalassaemic mice. *International Journal of Experimental Pathology*.

[64] Subramaniam V. N., McDonald C. J., Ostini L. (2012). Hepatic iron deposition does not predict extrahepatic iron loading in mouse models of hereditary hemochromatosis. *The American Journal of Pathology*.

[65] Brewer C., Otto-Duessel M., Wood R. I., Wood J. C. (2014). Sex differences and steroid modulation of cardiac iron in a mouse model of iron overload. *Translational Research*.

[66] Ramos E., Ruchala P., Goodnough J. B. (2012). Minihepcidins prevent iron overload in a hepcidin-deficient mouse model of severe hemochromatosis. *Blood*.

[67] Fleming R. E., Ponka P. (2012). Mechanisms of disease: iron overload in human disease. *The New England Journal of Medicine*.

[68] Cohen-Solal A., Leclercq C., Deray G. (2014). Iron deficiency: an emerging therapeutic target in heart failure. *Heart*.

[69] Pullarkat V. (2014). Iron toxicity in hematopoietic stem cell transplantation: strike while the iron is labile. *Acta Haematologica*.

[70] Pietrangelo A. (2010). Hereditary hemochromatosis: pathogenesis, diagnosis, and treatment. *Gastroenterology*.

[71] Leon M. B., Borer J. S., Bacharach S. L. (1979). Detection of early cardiac dysfunction in patients with severe beta-thalassemia and chronic iron overload. *The New England Journal of Medicine*.

[72] Camaschella C., Roetto A., de Gobbi M. (2002). Juvenile hemochromatosis. *Seminars in Hematology*.

[73] Gulati V., Harikrishnan P., Palaniswamy C., Aronow W. S., Jain D., Frishman W. H. (2014). Cardiac involvement in hemochromatosis. *Cardiology in Review*.

[74] Sebastiani G., Pantopoulos K. (2011). Disorders associated with systemic or local iron overload: from pathophysiology to clinical practice. *Metallomics*.

[75] Coates T. D. (2014). Physiology and pathophysiology of iron in hemoglobin-associated diseases. *Free Radical Biology and Medicine*.

[76] Pullarkat V. (2009). Objectives of iron chelation therapy in myelodysplastic syndromes: more than meets the eye?. *Blood*.

[77] Wood J. C. (2009). History and current impact of cardiac magnetic resonance imaging on the management of iron overload. *Circulation*.

[78] Anderson L. J., Holden S., Davis B. (2001). Cardiovascular T2-star (T2^*^) magnetic resonance for the early diagnosis of myocardial iron overload. *European Heart Journal*.

[79] Moussavi F., Ghasabeh M. A., Roodpeyma S. (2014). Optimal method for early detection of cardiac disorders in thalassemia major patients: magnetic resonance imaging or echocardiography?. *Blood Research*.

[80] Baksi A. J., Pennell D. J. (2014). Randomized controlled trials of iron chelators for the treatment of cardiac siderosis in thalassaemia major. *Frontiers in Pharmacology*.

[81] Brittenham G. M. (2011). Iron-chelating therapy for transfusional iron overload. *The New England Journal of Medicine*.

[82] Pennell D. J., Berdoukas V., Karagiorga M. (2006). Randomized controlled trial of deferiprone or deferoxamine in beta-thalassemia major patients with asymptomatic myocardial siderosis. *Blood*.

[83] Pepe A., Meloni A., Capra M. (2011). Deferasirox, deferiprone and desferrioxamine treatment in thalassemia major patients: cardiac iron and function comparison determined by quantitative magnetic resonance imaging. *Haematologica*.

[84] Fisher S. A., Brunskill S. J., Doree C., Gooding S., Chowdhury O., Roberts D. J. (2013). Desferrioxamine mesylate for managing transfusional iron overload in people with transfusion-dependent thalassaemia. *The Cochrane Database of Systematic Reviews*.

[85] Shalev O., Repka T., Goldfarb A. (1995). Deferiprone (L1) chelates pathologic iron deposits from membranes of intact thalassemic and sickle red blood cells both in vitro and in vivo. *Blood*.

[86] Kwiatkowski J. L. (2011). Real-world use of iron chelators. *Hematology/the Education Program of the American Society of Hematology*.

[87] Piga A., Fracchia S., Lai M. E. (2015). Deferasirox effect on renal haemodynamic parameters in patients with transfusion-dependent *β* thalassaemia. *British Journal of Haematology*.

[88] Kontoghiorghe C. N., Kolnagou A., Kontoghiorghes G. J. (2014). Antioxidant targeting by deferiprone in diseases related to oxidative damage. *Frontiers in Bioscience*.

[89] Zhou T., Ma Y., Kong X., Hider R. C. (2012). Design of iron chelators with therapeutic application. *Dalton Transactions*.

[90] Devanur L. D., Evans R. W., Evans P. J., Hider R. C. (2008). Chelator-facilitated removal of iron from transferrin: relevance to combined chelation therapy. *Biochemical Journal*.

[91] Tanner M. A., Galanello R., Dessi C. (2007). A randomized, placebo-controlled, double-blind trial of the effect of combined therapy with deferoxamine and deferiprone on myocardial iron in thalassemia major using cardiovascular magnetic resonance. *Circulation*.

[92] Tsushima R. G., Wickenden A. D., Bouchard R. A., Oudit G. Y., Liu P. P., Backx P. H. (1999). Modulation of iron uptake in heart by L-type Ca^2+^ channel modifiers: possible implications in iron overload. *Circulation Research*.

[93] Oudit G. Y., Trivieri M. G., Khaper N., Liu P. P., Backx P. H. (2006). Role of L-type Ca^2+^ channels in iron transport and iron-overload cardiomyopathy. *Journal of Molecular Medicine*.

[94] Kumfu S., Chattipakorn S., Chinda K., Fucharoen S., Chattipakorn N. (2012). T-type calcium channel blockade improves survival and cardiovascular function in thalassemic mice. *European Journal of Haematology*.

[95] Fernandes J. L., Sampaio E. F., Fertrin K. (2013). Amlodipine reduces cardiac iron overload in patients with thalassemia major: a pilot trial. *The American Journal of Medicine*.

[96] Shakoor A., Zahoor M., Sadaf A. (2014). Effect of L-type calcium channel blocker (amlodipine) on myocardial iron deposition in patients with thalassaemia with moderate-to-severe myocardial iron deposition: protocol for a randomised, controlled trial. *BMJ Open*.

[97] Jansová H., Macháček M., Wang Q. (2014). Comparison of various iron chelators and prochelators as protective agents against cardiomyocyte oxidative injury. *Free Radical Biology and Medicine*.

